# Supplementary material for: Alternative Pathway is Involved in Nitric Oxide-Enhanced Tolerance to Cadmium Stress in Barley Roots
Source: Plants (Basel). 2019 Nov 29;8(12):557. doi: 10.3390/plants8120557 (PMC6963264; doi:10.3390/plants8120557)
Supplement: Supplementary file 1 [file plants-08-00557-s001.pdf]

Supplemental materials.

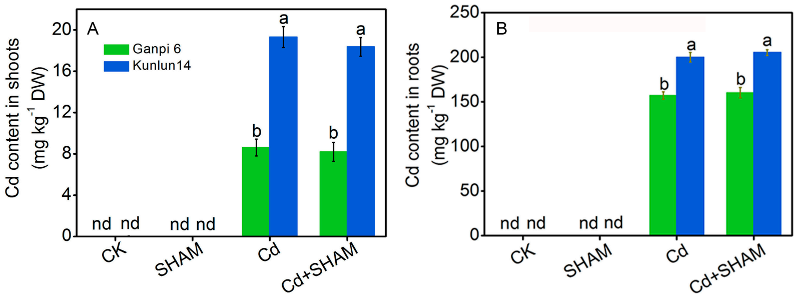

Supplementary Figure.1. Effect of SHAM on Cd content in shoots (A) and roots (B) of two barley varieties under Cd Stress. In the experiment, 150 μM Cd, 100 μM SHAM were used. Bars represent mean±SE (n=3), and different lower case letters represent significant difference at $P<0.05$. nd = not detectable.

Supplementary Table.1 Correlation coefficient between AP and the enzymatic activities, GSH, ASA, DHA, GSSG, NR, NOS in two barley roots.

	AP	SOD	CAT	APX	GSH	AsA	DHA	GSSG	GR	GPX	DHAR	MDHAR	NR	NOS
AP	1.0000	0.2043	0.1456	0.0596	0.1970	0.0848	-0.1164	0.0707	0.2181	0.1543	-0.3200	0.1335	-0.893*	-0.0878
SOD	0.2043	1.0000	0.2263	0.983**	-0.0346	0.4915	-0.2460	0.958**	-0.2425	0.951**	-0.912*	0.898*	-0.1912	-0.0613
POD	0.1431	-0.6299	-0.1062	-0.6051	0.4503	-0.0091	0.4583	-0.7635	0.7841	-0.4353	0.6999	-0.5568	-0.5216	-0.2576
CAT	0.1456	0.2263	1.0000	0.0726	-0.6643	0.7485	-0.7809	0.0961	0.2253	0.3264	0.1418	0.4231	-0.3886	0.6897
APX	0.0596	0.983**	0.0726	1.0000	0.0603	0.4331	-0.1523	0.953**	-0.2244	0.936**	-0.936**	0.879*	-0.1777	-0.1430
GSH	0.1970	-0.0346	-0.6643	0.0603	1.0000	-0.3307	0.946**	-0.1149	0.2644	-0.0795	-0.1525	-0.1972	-0.2893	-0.968**
AsA	0.0848	0.4915	0.7485	0.4331	-0.3307	1.0000	-0.6039	0.2883	0.5538	0.6675	-0.1010	0.7508	-0.7570	0.4459
DHA	-0.1164	-0.2460	-0.7809	-0.1523	0.946**	-0.6039	1.0000	-0.2520	0.0878	-0.3209	-0.0274	-0.4687	-0.0195	-0.945**
GSSG	0.0707	0.958**	0.0961	0.953**	-0.1149	0.2883	-0.2520	1.0000	-0.4797	0.872*	-0.956**	0.7907	0.0858	-0.0243
GR	0.2181	-0.2425	0.2253	-0.2244	0.2644	0.5538	0.0878	-0.4797	1.0000	-0.0067	0.4994	0.0242	-0.886*	-0.0261
GPX	0.1543	0.951**	0.3264	0.936**	-0.0795	0.6675	-0.3209	0.872*	-0.0067	1.0000	-0.7797	0.901*	-0.3780	0.0500
DHAR	-0.3200	-0.912*	0.1418	-0.936**	-0.1525	-0.1010	-0.0274	-0.956**	0.4994	-0.7797	1.0000	-0.6900	-0.1108	0.3033
MDHAR	0.1335	0.898*	0.4231	0.879*	-0.1972	0.7508	-0.4687	0.7907	0.0242	0.901*	-0.6900	1.0000	-0.3996	0.1758
NR	-0.893*	-0.1912	-0.3886	-0.1777	-0.2893	-0.7570	-0.0195	0.0858	-0.886*	-0.3780	-0.1108	-0.3996	1.0000	0.1105
NOS	-0.0878	-0.0613	0.6897	-0.1430	-0.968**	0.4459	-0.945**	-0.0243	-0.0261	0.0500	0.3033	0.1758	0.1105	1.0000
Kunlun14														
AP	1.0000	0.1619	0.0832	0.1341	-0.3379	0.1252	0.2414	0.1601	0.2078	0.0873	0.0226	0.0860	-0.918**	0.0262
SOD	0.1619	1.0000	0.3753	0.4633	-0.0931	0.4287	-0.4721	0.917*	0.953**	0.4568	-0.7198	0.884*	-0.3259	0.886*
POD	0.2040	-0.0257	0.1158	-0.3506	-0.2080	0.3997	0.5871	-0.2797	0.1974	0.5113	0.4113	0.4114	-0.5934	-0.3674
CAT	0.0832	0.3753	1.0000	0.2812	-0.0241	0.3712	0.4860	0.1905	0.3913	0.5882	0.2918	0.5430	-0.813*	0.0073
APX	0.1341	0.4633	0.2812	1.0000	0.7034	-0.4656	-0.3892	0.6472	0.2289	-0.3366	-0.4707	0.2963	0.0475	0.4541
GSH	-0.3379	-0.0931	-0.0241	0.7034	1.0000	-0.883*	-0.2094	0.2246	-0.3343	-0.7287	-0.0700	-0.1429	0.3671	-0.0269
AsA	0.1252	0.4287	0.3712	-0.4656	-0.883*	1.0000	0.2706	0.0560	0.6615	0.956**	0.0147	0.5683	-0.6893	0.1903
DHA	0.2414	-0.4721	0.4860	-0.3892	-0.2094	0.2706	1.0000	-0.7139	-0.2846	0.4276	0.930**	-0.0635	-0.6745	-0.817*
GSSG	0.1601	0.917*	0.1905	0.6472	0.2246	0.0560	-0.7139	1.0000	0.7608	0.0730	-0.849*	0.6846	0.0198	0.943**
GR	0.2078	0.953**	0.3913	0.2289	-0.3343	0.6615	-0.2846	0.7608	1.0000	0.6783	-0.5798	0.935**	-0.4992	0.7667
GPX	0.0873	0.4568	0.5882	-0.3366	-0.7287	0.956**	0.4276	0.0730	0.6783	1.0000	0.1419	0.6822	-0.863*	0.1115
DHAR	0.0226	-0.7198	0.2918	-0.4707	-0.0700	0.0147	0.930**	-0.849*	-0.5798	0.1419	1.0000	-0.3695	-0.3796	-0.939**
MDHAR	0.0860	0.884*	0.5430	0.2963	-0.1429	0.5683	-0.0635	0.6846	0.935**	0.6822	-0.3695	1.0000	-0.6537	0.5818
NR	-0.918**	-0.3259	-0.813*	0.0475	0.3671	-0.6893	-0.6745	0.0198	-0.4992	-0.863*	-0.3796	-0.6537	1.0000	0.1305
NOS	0.0262	0.886*	0.0073	0.4541	-0.0269	0.1903	-0.817*	0.943**	0.7667	0.1115	-0.939**	0.5818	0.1305	1.0000

* and.** represent P<0.05 and 0.01,respectively.
